# Surgical Management of a Complex High Hepatic Duct Injury: A Case Report

**DOI:** 10.7759/cureus.80313

**Published:** 2025-03-09

**Authors:** Audrey Brecher, Grace E Trello, Jordan Perkins, Chintalapati Varma, Mustafa Nazzal

**Affiliations:** 1 Department of Surgery, Saint Louis University School of Medicine, Saint Louis, USA; 2 Department of Surgery, Saint Louis University Hospital, Saint Louis, USA

**Keywords:** bile duct injury, cholecystitis, kasai portoenterostomy, laparoscopic cholecystectomy, strasberg-bismuth class e4

## Abstract

This is a case of a patient who underwent an elective laparoscopic cholecystectomy after previously presenting with acute cholecystitis. His operation was complicated by a high common hepatic duct (CHD) injury requiring complex surgical repair. This case highlights the importance of the early recognition of this type of injury and the utility of a Kasai portoenterostomy as an operative repair in high CHD injuries in the absence of an extrahepatic duct viable for repair.

## Introduction

Cholecystectomy remains one of the most performed surgeries worldwide. The standard surgical approach has shifted from open to laparoscopic, with a resulting increase in injury to the biliary tree [1] that has been attributed to the incorrect identification of the biliary anatomy [2]. The Critical View of Safety (CVS) is a well-known technique that assists in ensuring the proper identification of the cystic artery and duct prior to their division [3]. This CVS was created with the intention of reducing biliary injuries by encouraging systematic identification of cystic structures prior to gallbladder removal [4]. Other techniques, like the use of intraoperative cholangiograms, can help identify biliary anatomy in difficult cases. Alternatively, surgeons can elect to proceed with a subtotal cholecystectomy or to convert to an open procedure if there is concern about the biliary anatomy. However, most injuries to the biliary system are not noted at the time of operation, delaying timely diagnosis.

The Strasberg-Bismuth classification is the commonly used system for describing injury and stricture to the biliary system [5] based on the relationship to the junction of the hepatic biliary system. This system incorporates consideration of the distance of the injury, more specifically the stricture, from the confluence of the right and left bile ducts and bile duct injury patterns, such as bile leaks [6]. For example, cystic duct leaks or leaks from small ducts in the liver bed are classified as type A biliary duct injury, the least severe. Conversely, stricture to an aberrant right hepatic duct and the common hepatic duct is considered the most severe injury and is categorized as type E5.

While injury to the biliary system during laparoscopic cholecystectomy is rare, estimated at 0.7% of all cases [1], it remains a feared complication due to the associated morbidity and mortality. The specific repair of these injuries is multifactorial and based on the time at which the injury was recognized as well as the anatomy of the injury. Furthermore, there are very limited approaches to this kind of problem; most often, the option is between percutaneous transhepatic biliary drainage and wide local drainage. We present a case of a patient with a Strasberg-Bismuth (class E4) biliary duct injury, above the level of the hepatic biliary system confluence, ultimately requiring a portoenterostomy (PE). This procedure, often referred to as the Kasai procedure, is a mainstay treatment for biliary atresia. There are a few cases in the literature of adults who have undergone PE and little evidence regarding postoperative outcomes in adults who have undergone the procedure.

This case was previously presented as a meeting oral presentation at the 2023 Missouri American College of Surgeons Annual Meeting on April 20, 2024.

## Case presentation

Preoperative course

A 66-year-old male patient with a past medical history significant for hyperparathyroidism (status postparathyroidectomy) and 4 cm thoracic aortic aneurysm presented to an outside hospital (OSH) with a work-up consistent with acute cholecystitis. At that time, subsegmental pulmonary emboli were also incidentally discovered and surgery was delayed so he could be started on anticoagulation. Three months later, the scheduled laparoscopic surgery was converted to an open cholecystectomy due to significant adhesive disease. The patient’s early postoperative course was complicated by atrial fibrillation with rapid ventricular response and abdominal distension. CT imaging was obtained and revealed a perihepatic fluid collection. Aspiration of the fluid collection was then performed, with over a liter of bilious output. Subsequent endoscopic retrograde cholangiopancreatography was performed and was notable for its inability to cannulate the common bile duct. He then underwent diagnostic laparotomy, washout, and Jackson-Pratt (JP) drain placement into the gallbladder fossa on postoperative day nine. The patient was then transferred to a tertiary care center for hepatobiliary surgical evaluation given the high suspicion for a common bile duct injury.

**Figure 1:**
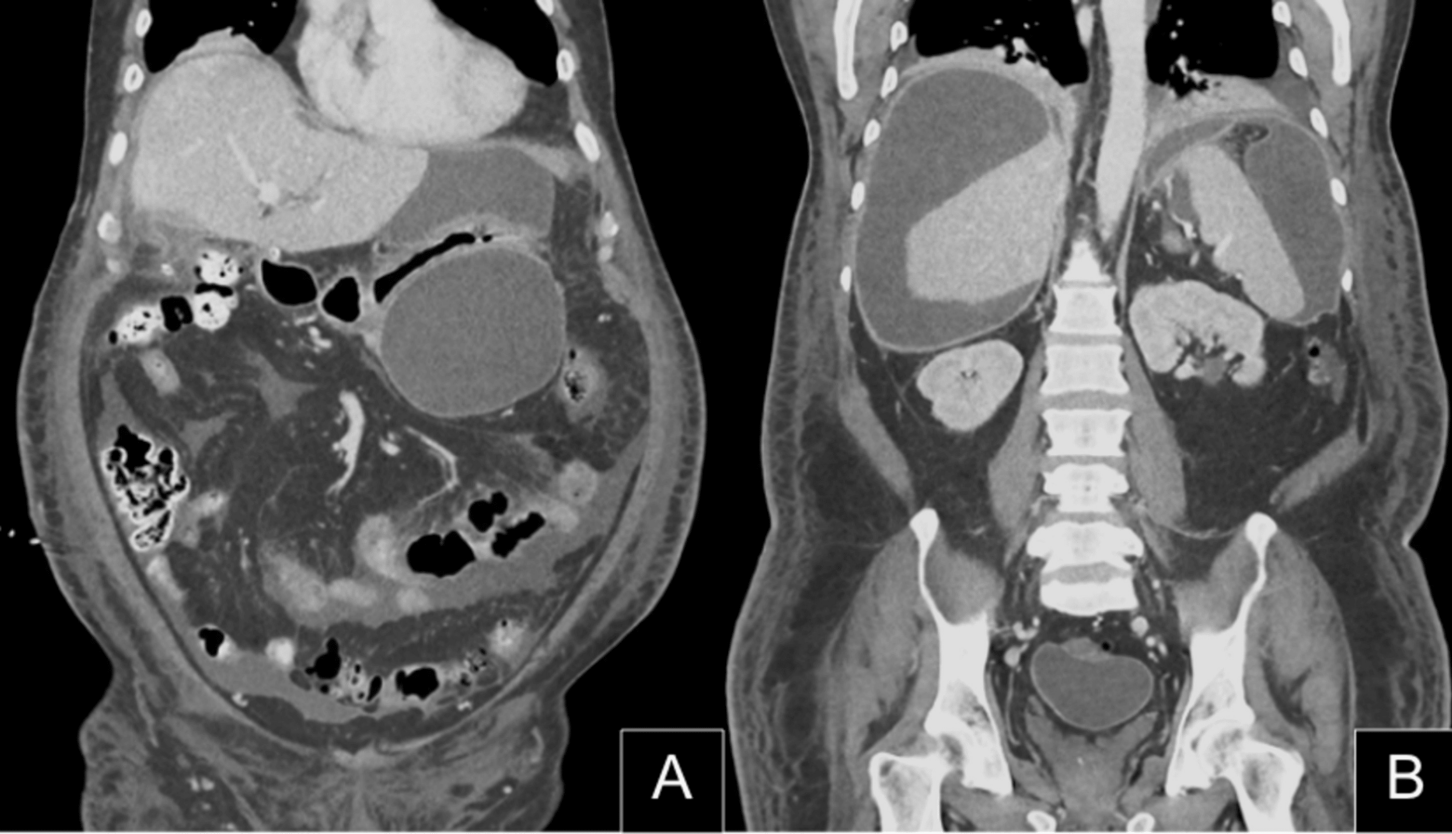
Representative coronal images from the initial CT scan (A) Free fluid and several loculated fluid collections are noted around the stomach. (B) Free fluid and several loculated fluid collections are noted around the liver and spleen as well. Small-volume reactive bilateral pleural effusions are also seen as well as pneumobilia.

**Figure 2:**
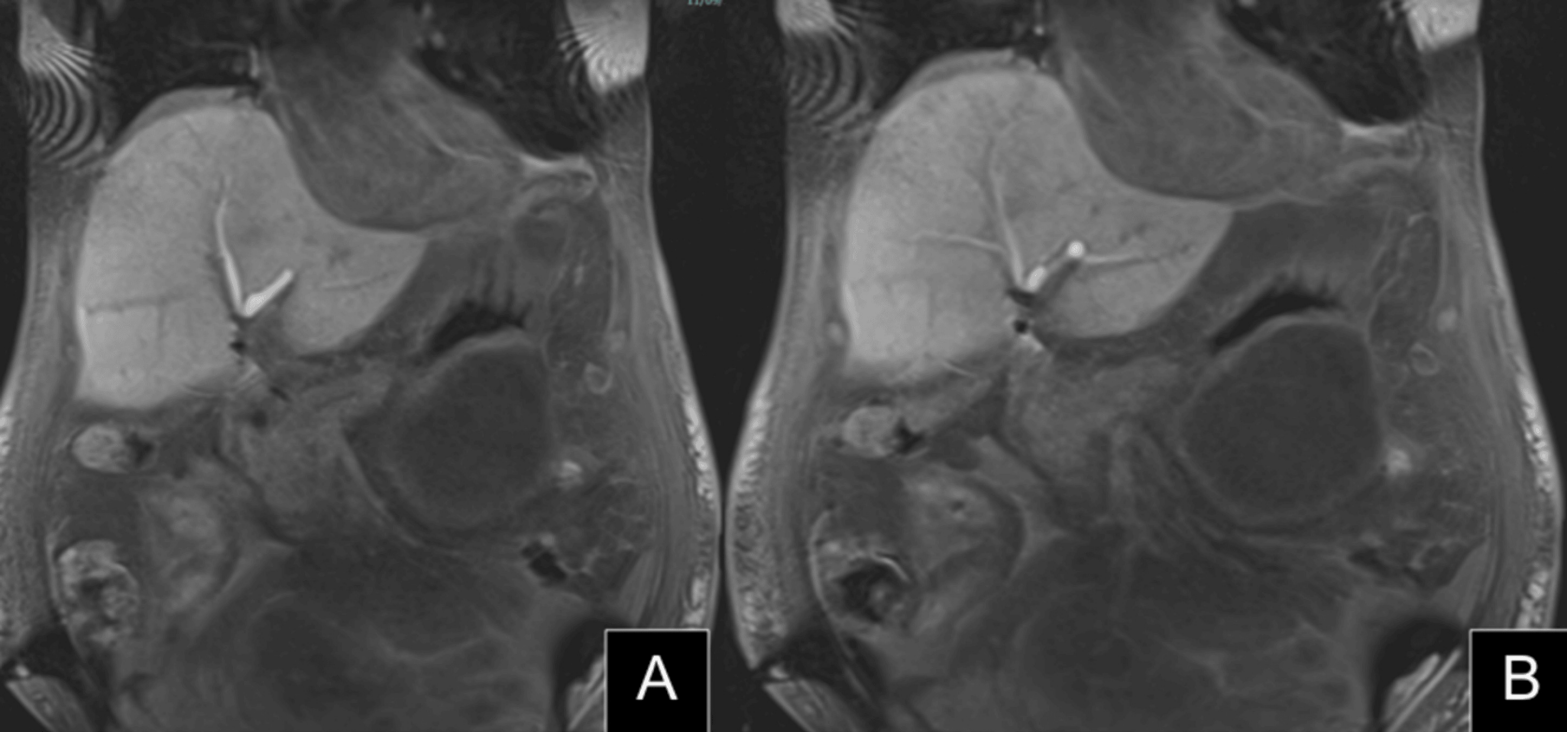
Representative coronal images from the initial MRI scan (A) Redemonstration of previous fluid collections. (B) A metallic artifact is seen at the junction of the right and left hepatic ducts with the inability to visualize the common hepatic or common bile duct.

**Figure 3:**
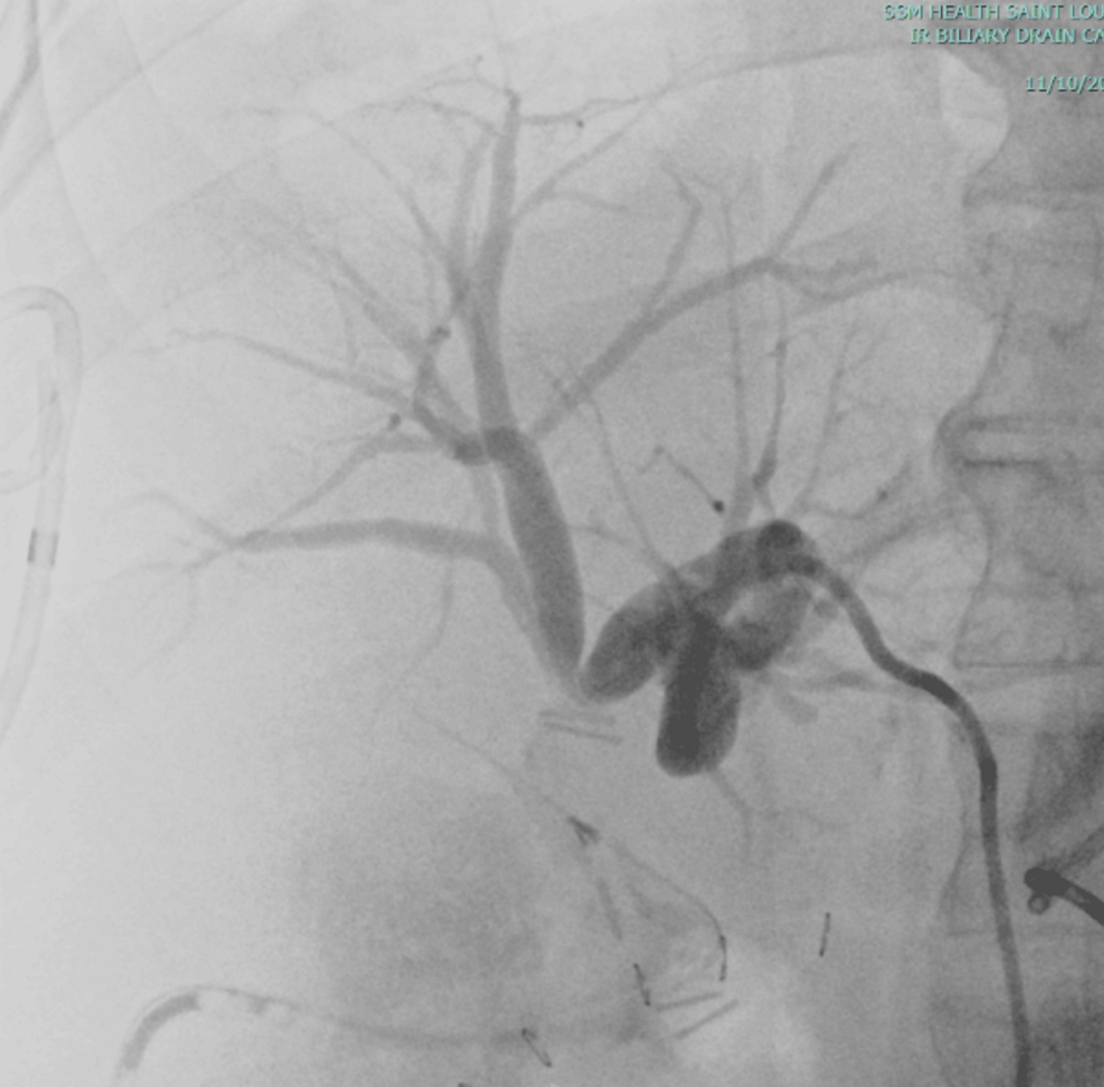
Representative image from the percutaneous transhepatic drain placement with a cholangiogram Two metallic clips are again seen at the junction of the right and left hepatic ducts. Unable to cannulate the right biliary system.

On arrival, the patient was alert and reported intermittent abdominal pain that had been responsive to medications. Abdominal examination was notable for mild tenderness to palpation and distension. A JP drain from the index operation showed bilious drainage. Laboratory results were notable for a total bilirubin of 1.8 (direct 1.1) and a leukocytosis. A CT scan (Figure 1) to rule out any concomitant vascular injury was performed and showed no vascular injuries. However, this CT image was notable for perigastric, perisplenic, and perihepatic fluid collections concerning for bilomas. Magnetic resonance cholangiopancreatography (MRCP) (Figure 2) revealed a metallic artifact at the level of the common hepatic duct with no visible common bile duct distal to this level. Lack of visualization of the common hepatic duct and common bile duct was suggestive of injury at the level of the proximal common hepatic duct. In order to further delineate the injury, study the proximal biliary system, and control the bile leak, interventional radiology was consulted, and they placed drains in the perihepatic and perigastric fluid collections. A cholangiogram redemonstrated a metallic artifact at the junction of the right and left biliary system (Figure 3), and a percutaneous transhepatic biliary drain (PTBD) was left within the left hepatic system. The right biliary system was not able to be cannulated but was visualized from the left system. Fluid cultures from drains grew *Enterobacter*, and the patient was subsequently started on IV antibiotics. Given the delayed diagnosis of biliary injury and intra-abdominal infections, definitive surgical intervention was deferred. He was discharged with drains, antibiotics, and daily IV fluid boluses given the high output from the drains and PTBD. His outpatient course was then complicated by an acute deep vein thrombosis (DVT) requiring hospital admission. Imaging revealed persistent fluid collections. Two additional drains were placed, and he was discharged on therapeutic low-molecular-weight heparin. He was again admitted two days before his scheduled surgery due to failure to thrive. A feeding tube was placed, and he was noted to have bilateral lower extremity non-occlusive DVTs with subsequent inferior vena cava (IVC) filter placement.

Operative details: exploratory laparotomy with hepaticojejunostomy

The abdomen was opened via a J-shaped incision via a right subcostal incision with a midline extension. After extensive lysis of adhesions, the gallbladder fossa was visualized, and two sets of clips were identified. One set was extremely proximal inside the hilar plate of the liver, and another set was found on the distal bile duct. There was active bile extravasation at the site of the proximal clips with necrosis of the distal aspects of the left and right hepatic ducts. There did not appear to be a common trunk between the two hepatic ducts consistent with a Strasberg-Bismuth E4 injury.

**Figure 4:**
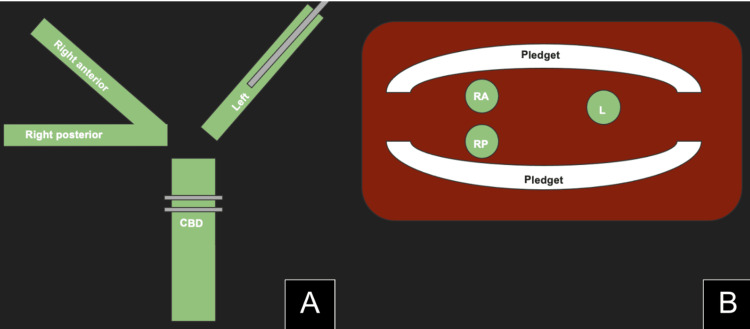
Diagram representing the pledget placement (A) Interposing pledget sutures (in addition to 3-0 suture) were used around the common bile duct at the branching point of the right anterior (RA), right posterior (RP), and left main hepatic (L) ducts to create anastomosis. (B) A cross-sectional view of pledgets placed around RA, RP, and L ducts. CBD: common bile duct Image credits: Dr. Jordan Perkins

Given the absence of any extrahepatic common hepatic duct and multiple hepatic duct ostia at the hilar plate (Figure 4A), the decision was made to perform a Roux-en-Y PE. The Roux limb was placed in a retrocolic fashion, and a 2-cm enterotomy was created. Using 3/0 polydioxanone sutures (PDS), the enterotomy was approximated to the hilar plate surrounding the cut biliary ducts. In order to get an appropriate seal to the liver, interposing pledgets were also used for the PE to the hilar plate and liver tissue around the bile ducts (Figure 4B) to prevent cutting through the liver tissue. The previously placed left side PTBD was utilized as a stent that transverses the PE anastomosis. An omental patch was created around the anastomosis, and a JP drain was introduced under the anastomosis.

Postoperative course

The patient tolerated the procedure well, but nutrition remained a consistent issue. He was discharged on postoperative day 13 with enteral nutrition using a feeding nasoduodenal tube as well as oral nutrition. Drains in place at discharge included a perisplenic drain, PTBD, and JP drain around the PE anastomosis. His enteral tube and drains were removed sequentially over several weeks. The PTBD was utilized for a pull cholangiogram, which demonstrated a patent PE and the absence of a leak. Eventually, his PTBD was removed after being capped for a few weeks.

## Discussion

While injury to the common bile duct system is rare, this case emphasizes the highly associated morbidity. Studies have shown that timely transfer to a center with specialized hepatobiliary service improves outcomes [7-9]. In this patient’s case, his definitive operation was delayed to obtain adequate source control and drainage of the biliary system and treatment of infection to allow acute inflammation to subside prior to surgery. The biliary system was studied through endoscopic retrograde cholangiopancreatography (ERCP), percutaneous transhepatic cholangiogram, and MRCP with a double dose of Eovist contrast (Bayer AG, Leverkusen, Germany) to understand the biliary anatomy and help the operative planning in such a complex case.

This case provides support for delayed surgical repair of Strasberg-Bismuth E4 common bile duct injuries with PE to achieve better outcomes with a permanent solution compared to percutaneous transhepatic biliary drainage or wide local drainage, both of which would have been temporary solutions. In this case, two months passed between the time of the initial injury and the PE repair. Multiple studies suggest that delayed surgical repair of the injury results in fewer complications in the long term such as biliary stricture formation [10,11]. The case report by Hristov et al. demonstrates the benefit of early surgical intervention for drain placement followed by delayed repair of the bile duct injury. As a result, this patient’s prognosis would have been improved by prompt drain placement, but his surgical management with delayed PE has been supported in the literature.

PE as a first-line treatment in the management of Strasberg-Bismuth E4 injuries is not generally supported by the literature at this time. Roux-en-Y hepaticojejunostomy continues to be the mainstay of treatment for biliary trauma involving transection of the common bile duct [12]. However, PE may become indicated when needed as a “salvage” procedure in select cases such as the one described in this report where there is no extrahepatic duct for anastomosis, or in the presence of multiple small ducts or intense fibrosis and inflammation [13]. In such cases, hepaticojejunostomy may not be feasible and PE may preferably be performed.

## Conclusions

The patient presented in this case report has a complex and high bile duct injury. He underwent a PE procedure, which is typically performed in the setting of biliary atresia in pediatric populations. While rare, injuries to the biliary tree during cholecystectomies are a highly morbid complication, and this case highlights the complexity of the operative repair and also the issues that arise in the preoperative and postoperative stages of care. A multidisciplinary approach with careful preoperative planning is integral in the management of patients with these types of complex injuries for the best prognosis.
